# The association between bone density of lumbar spines and different daily protein intake in different renal function

**DOI:** 10.1080/0886022X.2023.2298080

**Published:** 2024-01-08

**Authors:** Chia-Lin Lee, Kun-Hui Chen, Wei‑Ju Liu, Ching-Hsien Chen, Shang-Feng Tsai

**Affiliations:** a Department of Post-Baccalaureate Medicine, College of Medicine, National Chung Hsing University, Taichung, Taiwan; b School of Medicine, National Yang-Ming University, Taipei, Taiwan; c Division of Endocrinology and Metabolism, Department of Internal Medicine, Taichung Veterans General Hospital, Taichung, Taiwan; d Intelligent data mining laboratory, Department of Medical Research, Taichung Veterans General Hospital, Taichung, Taiwan; e Department of Public Health, College of Public Health, China Medical University, Taichung, Taiwan; f Department of Orthopedic Surgery, Taichung Veterans General Hospital, Taichung, Taiwan; g Department of Computer Science & Information Engineering, College of Computing and Informatics, Providence University, Taichung, Taiwan; h Divisions of Nephrology and Pulmonary, Critical Care and Sleep Medicine, Department of Internal Medicine, University of California at Davis, Davis, CA, USA; i Division of Nephrology, Department of Internal Medicine, Taichung Veterans General Hospital, Taichung, Taiwan; j Department of Life Science, Tunghai University, Taichung, Taiwan

**Keywords:** Bone density, lumbar spine, chronic kidney disease, protein diet, osteoporosis

## Abstract

**Background:**

Low protein intake (LPI) has been suggested as a treatment for chronic kidney disease (CKD). However, protein intake is essential for bone health.

**Methods:**

We studied the database of the National Health and Nutrition Examination Survey, 2005–2010. Basic variables, metabolic diseases, and bone density of different femoral areas were stratified into four subgroups according to different protein intake (DPI) (that is, <0.8, 0.8–1.0, 1.0–1.2, and >1.2 g/kg/day).

**Results:**

Significant differences were found among all lumbar area bone mineral density (BMD) and T-scores (*p* < 0.0001). There was an apparent trend between a decreasing BMD in the CKD groups with increasing DPI in all single lumbar spines (L1, L2, L3, and L4) and all L spines (L1-L4). Compared with DPI (0.8–1.0 g/day/kg), higher risks of osteoporosis were noticed in the subgroup of >1.2 g/day/kg over L2 (relative risk (RR)=1.326, 95% confidence interval (CI)=1.062–1.656), subgroup >1.2 g/day/kg over L3 (RR = 1.31, 95%CI = 1.057–1.622), subgroup <0.8 g/day/kg over L4 (RR = 1.276, 95%CI = 1.015–1.605), subgroup <0.8 g/day/kg over all L spines (RR = 11.275, 95%CI = 1.051–1.548), and subgroup >1.2 g/day/kg over all L spines (RR = 0.333, 95%CI = 1.098–1.618). However, a higher risk of osteoporosis was observed only in the non-CKD group. There was an apparent trend of higher DPI coexisting with lower BMD and T scores in patients with CKD. For osteoporosis (reference:0.8–1.0 g/day/kg), lower (<0.8 g/day/kg) or higher DPI (>1.2 g/day/kg) was associated with higher risks in the non-CKD group, but not in the CKD group.

**Conclusions:**

In the CKD group, LPI for renal protection was safe without threatening L spine bone density and without causing a higher risk of osteoporosis.

## KEY LEARNING POINTS

A low-protein diet should be encouraged in patients with CKD, but protein is essential for bone health. In this study, we showed that a low-protein diet did not affect lumbar bone density. Therefore, in the care of CKD, a low-protein diet is beneficial for renal function and without harm to lumbar bone health.

## Introduction

Osteoporosis is defined by bone mineral density (BMD), which is an essential determinant of bone strength and, hence, the risk of bone fracture [1]. In osteoporosis, fragility fractures occur spontaneously or after minor trauma. Reduced bone mineral density (BMD) is a major risk factor for fragility fractures. Protein diets affect bone strength as proteins constitute about half of the bone volume and 1/3 of the bone mass [2]. The collagen and noncollagen forms of proteins can provide a structural matrix. Proper protein diets are essential for maintaining bone mass. A meta-analysis of adults showed that protein intake is one of the primary anabolic stimuli for the biosynthesis of muscle proteins [3]. Therefore, the current Recommended Dietary Allowance (RDA) of protein intake for adult is 0.8 g/kg/day [4]. However, there are still some disadvantages to using proteins involved in bone formation. For example, proteins may be metabolized to acids, which under certain conditions can lead to metabolic acidosis. Acidosis further worsens the function of osteoblasts and extends the life of osteoclasts [5]. Therefore, protein intake that is too high is not recommended for bone health. To date, the association between dietary protein intake and bone metabolism is still lacking in the general population.

Chronic kidney disease (CKD) is a heterogeneous disorder characterized by impaired kidney structure and function with a number of presentations and outcomes related to the underlying cause and disease severity [6,7]. All patients have nephron loss, with the remaining viable glomeruli showing hyperfiltration [8]. Glomerular hyperfiltration further impairs viable nephrons, leading to a vicious cycle of dysfunction. In 1996, Barry Brenner proposed a theory called ‘the hyperfiltration theory: a paradigm shift in nephrology’ [9]. He postulated that, in most CKD, glomerular hyperfiltration exacerbates the progression of renal damage [9]. Therefore, treatments for controlling glomerular hyperfiltration should include the following: aggressive control of blood pressure, low salt diet [10], low animal protein diet [10,11], the use of renin-angiotensin system inhibitors (RASi) [12–17], sodium-glucose cotransporter-2 inhibitors [18], endothelin-1 antagonist [19], finerenone [20,21] and glucagon-like peptide 1 receptor agonists [22]. A high animal protein diet increases intraglomerular pressure *via* afferent arteriolar vasodilation, leading to CKD [23]. Therefore, low-protein diets (0.6–0.8 g/kg/day) are routinely recommended for CKD patients to minimize glomerular hyperfiltration [24–27]. Three recent studies further support the benefit of a low-protein diet in CKD care to obtain better renal function [11,28,29]. Low low-protein diet is also recommended in the Kidney Disease: Improving Global Outcomes (KDIGO) guideline [30] to curtail the progression of CKD. However, very few studies have investigated whether low-protein diet intervention threatens bone health in CKD patients. The association between different amounts of daily protein intake (DPI) and bone health has not been studied in patients with CKD, with the exception of our previous study in the hip area [31].

CKD has a detrimental impact on bone strength. Nonetheless, the osteoporotic manifestations observed in CKD patients remain inadequately elucidated. In a prior investigation employing high-resolution peripheral quantitative computed tomography (HR-pQCT) to assess the three-dimensional microarchitecture of bone, it was demonstrated that individuals at CKD stage 5 D exhibited notable tibial microstructural impairments in comparison to those at CKD stage 4–5 [32]. Meanwhile, patients with stage-3 CKD start to develop secondary hyperparathyroidism (hypocalcemia, hyperphosphatemia, and hyperparathyroidism), low serum levels of active vitamin D, more calcium-containing medications with meals (for phosphate binders), and metabolic acidosis [33]. As renal function deteriorated, the condition worsened. All of these conditions in CKD can affect bone health. From a cross-sectional study in 2020 [34], the results showed that femoral BMD rather than lumbar BMD was positively correlated with the eGFR in the CKD population. In our previous study, we found that a low DPI is safe for bone health in the femoral neck area in patients with CKD **[**31]. The peak adult bone mass, rate of bone loss, and artifacts were not the same throughout the skeleton. Therefore, in the present study, we analyzed bone density over the lumbar (L) spine among different DPI with or without CKD.

## Subjects and methods

### Study population and data collection

#### National Health and Nutrition Examination survey (NHANES)

The National Health and Nutrition Examination Survey (NHANES) is a health-related program conducted periodically by the Centers for Disease Control (CDC) and Prevention’s National Center for Health Statistics (NCHS), which released this dataset. The Research Ethics Review Board at the NCHS approved the survey protocol, all participants, or proxies, and provided written informed consent. This large ongoing dietary survey was conducted to assess the health and nutritional status of community-dwelling individuals in the U.S. Nutritional status data are very detailed and have been extensively investigated in many published studies. The examinations included laboratory data, questionnaires on health and nutrition, and anthropometric measurements. All participants completed in-home interviews. We analyzed participants in the NHANES from 2005 to 2010. Participants were only included in our study under the following criteria: >18 years of age with data on renal function (estimated glomerular filtration rate) (eGFR), and had complete data with respect to anthropometric measurements, questionnaires, and laboratory examinations.

##### Definition for protein diet

The current RDA of protein intake is 0.8 g protein/kg BW/day for adults [35]. For an elderly subject, higher intakes have been recommended, like 1.0–1.2 to 1.2–1.5 g/kg/day to maintain muscle functions [36]. For CKD patients, restricted daily protein intake is approximately 0.6–0.8 g/kg/day [37–39]. For CKD patients, most guidelines, including KDIGO, recommend a restricted protein diet such as a low protein diet (0.6 g/kg/day) or a very low protein diet (0.2 g/kg/day) [40]. Thus, we stratified our subject population based on their daily protein intake (DPI) into 4 groups: (a) <0.8 g/kg/day, (b) 0.8–1.0 g/kg/day, (c) 1.0–1.2 g/kg/day, and (d) ≥1.2 g/kg/day. This grouping scheme was also used in our previous related study (on the impact of DPI on hip fractures in patients with and without CKD from NHANES) [31]. DPI information was collected by an interviewer administering 24-h recalls using the U.S. Department of Agriculture Automated Multiple-Pass Method. This is a 5-step procedure to quantify 24-h food and beverage intakes [36].

##### Definition of osteoporosis

The study subjects were evaluated using dual-energy X-ray absorptiometry (DXA) for BMD (g/cm^2^). BMDs of the lumbar (Ls) spines, including L1, L2, L3, L4, and all L spines, were evaluated using DXA (Hologic Inc., Bedford, MA, USA). All measurements were performed according to the standard procedures [37,40]. In the field of osteoporosis, according to the International Society for Clinical Densitometry (ISCD), clinicians use combined L1-L4 for spine BMD measurement. However, BMD and T score data in all lumbar spines should be initially presented as individual vertebrae (from L1 to L4). The major reason is that the presentation of individual vertebrae to see is there any more than a 1.0 T-score difference between the vertebrae in question and the adjacent vertebrae. If the T-score difference is greater than 1.0, the vertebral data may be excluded from the analysis. Therefore, we presented all individual lumbar spine BMD and T-score data.

Quality control was routinely conducted using all the DXA machines. According to the measured BMD and World Health Organization criteria [38], those patients with T scores between −1.0 and −2.5 were defined as having osteopenia. Moreover, those with T scores < −2.5 were defined as having osteoporosis. Both CKD and non-CKD groups were diagnosed according to the above criteria [38].

NHANES performed a continuous, nationwide representative health survey of civilian, non-institutionalized US people and collected data on about 5000 persons per year from interviews, physical examinations, and medical data including ‘bone densitometry’. In 1999, NHANES began performing DXA whole-body measurements on survey subjects aged 8 years and older in three mobile examination centers [41]. Less than 10% of participants did not have DXA data because of their physiological state (pregnant women or amputations).

##### Other data collection

Baseline variables according to the four groups of DPI included the following: age, sex, body mass index (BMI) (kg/m^2^), glycated hemoglobin (HbA1c) (%), eGFR according to the Modification of Diet in Renal Disease (MDRD) (ml/min/1.732 m^2^), systolic blood pressure (SBP) (mmHg), diastolic blood pressure (DBP) (mmHg), total cholesterol (mg/dl), high-density lipoprotein (mg/dl), triglyceride (mg/dl), and fasting plasma glucose (mg/dl). HbA1c levels were measured using boronate affinity high-performance liquid chromatography (CLC385 TM, Primus, Kansas City, Mo., USA). The equation of eGFR (ml/min/1.732 m^2^) according to MDRD was as follows: eGFR = 186 × serum creatinine (mg/dl) ^−1.154^ × years ^−0.203^ × (0.742, if female) × (1.210, if African American) [42]. The MDRD formula (instead of the Cockcroft and Gault formula or Chronic Kidney Disease Epidemiology Collaboration (CKD-EPI) formula) was chosen because of its higher accuracy for diabetic patients (1555 cases in this study) with impaired renal function [43]. CKD-EPI was first used in 2009. Our population covered the period mostly before 2009 [44–49]. MDRD for diagnosing CKD has also been published in journals with high impact factor [50,51]. Therefore, CKD was defined as eGFR < 60 mL/min/1.73 m^2^ according to the MDRD equation. Data from the CKD-EPI definition are also presented in the supplementary data. This study was approved by the Human Research Review Committee of Taichung Veterans General Hospital (approval number CE19051B). All methods were performed in accordance with the relevant guidelines and regulations, including a statement in the Methods section regarding this effect.

### Methods

We conducted this retrospective study of participants in the NHANES from 2005 to 2010. Participants were only included in our study if they had sufficient renal function data, DXA values (BDM and T score), and completed nutrition questionnaires. Specifically, for the studied subjects, the basic variables, metabolic diseases, and bone density of different femoral areas were stratified into four subgroups according to different levels of protein diet of dietary protein intake (DPI) (that is, <0.8, 0.8–1.00, 1.0–1.2, and >1.2 g/kg/day). We then compared the differential femoral areas among these subgroups for both the CKD and non-CKD subjects.

### Statistical analyses

For continuous variables (e.g. BMD and T-score), data are presented as the mean ± SEM. BMD, T score, and osteoporosis from all individual L spines were presented according to different DPI with or without CKD. All *p* values for comparisons were two-sided and considered significant at *p* < 0.05. In addition, 95% confidence intervals (CI) were calculated. For other continuous variables, we presented them as mean (95% CI). Because of the complex survey design of the NHANES study (e.g. a complex survey designed with stratification, clustering, and/or unequal weighting), the usual estimates were inappropriate. The analyses were weight-adjusted to represent the U.S population. The key concept of weighting in the NHNAES is to account for the complex survey design (including oversampling), survey non-response, and post-stratification. Weighted data were calculated according to online analytic guidelines (NHNES: Analytic Guidelines, 2011–2014 and 2015–2016) [25]. Some examples of papers with reweighted NHANES data can be seen [52,53]. Analysis of variance (ANOVA) was used to examine significant differences in baseline demographics and characteristics across different levels of protein intake. The sample-weighted AVOVA test was performed using the SAS SURVEYREG Procedure according to the user’s instructions. Subgroups of DPI were: (a) <0.8 g/kg/day, (b) 0.8–1.0 g/kg/day, (c) 1.0–1.2 g/kg/day, and (d) >1.2 g/kg/day. To overcome possible confounding factors, we performed adjusted and weighted tests using the SURVEYREG Procedure to compare the BMD and T-score levels among different subgroups of protein intake after adjusting for age, sex, energy intake, and BW. We also related lumbar bone density with the clinical outcome (risk of osteoporosis) (data presented as relative risk (RR), 95% CI) according to the cutoff criteria in a previous study [54]. Subgroup analyses were further performed for elderly patients and those without CKD, daily calcium intake, daily phosphorus intake, blood calcium concentration, blood phosphorus concentration, daily vitamin D intake, and blood vitamin D concentration. In addition, we used weighted logistic regression analyses to compare osteoporosis risk across different levels of protein intake using the SAS SURVEYLOGISTIC Procedure. All analyses were conducted using Statistical Analysis System survey procedures (SAS version 9.4, 2013, Cary, NC, USA).

## Results

### Baseline characteristics of population divided by different protein intake

**Figure 1.**
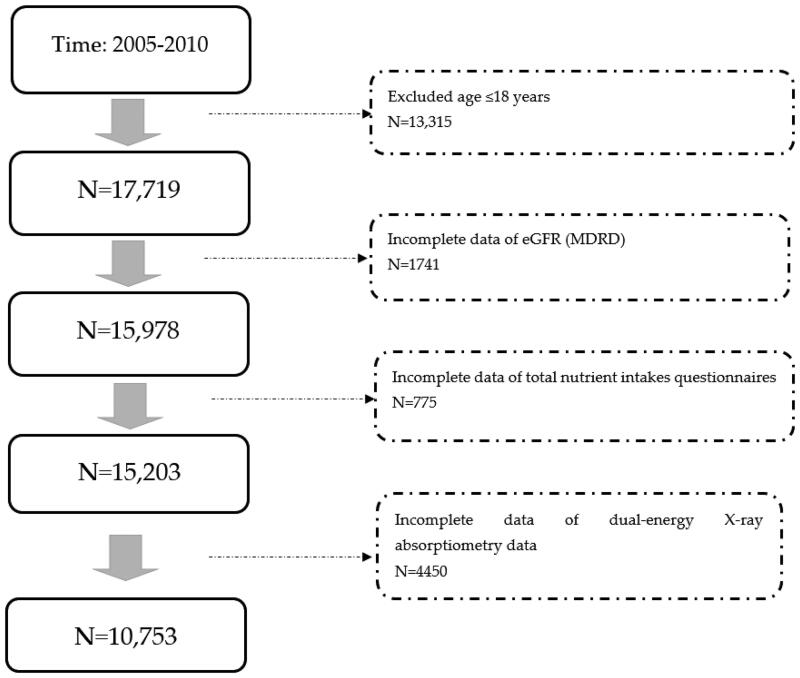
Algorithm for the selection of participants.

Initially, 31,034 participants were included in the study. After exclusion (age≦18y/o, no eGFR data, incomplete data from total nutrient intake questionnaires, and no data on nutrient intake and bone density), 10,753 participants were analyzed (Figure 1). Their mean age was relatively young (44.3 y/o) and most were healthy (Table 1). However, many of them were obese (27.89 kg/m^2^ of BMI). As for metabolic syndrome, few had hypertension (122.35 mmHg of mean SBP, 70.87 mmHg; DBP), their total cholesterol was 197.03 mg/dl and only 10.9% had DM. Few patients had renal dysfunction (96.69 mL/min/1.732 m^2^ of mean eGFR), and only 5.93% of patients had CKD. The mean protein intake was 1.1 g/kg per day. People who consumed more proteins were more likely to be younger, male, with lower BW and lower BMI (all *p* < 0.0001). They also had fewer CKD (based on both the MDRD and CKD-EPI formulas), higher eGFR, fewer DM (less fasting glucose and HbA1c), lower SBP, and greater calorie intake (all *p* < 0.0001). Among all four subgroups of protein intake, we found no significant inter-subgroup differences in all lumbar area BMD and T-scores (*p* < 0.0001).

**Table 1.** Baseline characteristics of population divided by different protein intake.

	Daily protein intake per day, g/kg					
Variables	Overall	<0.8	0.8–1.0	1.0–1.2	>1.2	*p*-value
N	10753	3849	1936	1516	3452	
Age (years old)	44.25(43.61–44.89)	46.82(46.14–47.5)	45.67(44.79–46.54)	44.69(43.53–45.85)	40.82(39.96–41.68)	<0.0001
Male, n (%)	5398(49.37)	1523(37.82)	923(46.15)	812(52.3)	2140(60.9)	<0.0001
Body weight (kg)	80(79.43–80.57)	85.18(84.38–85.98)	81.19(80.12–82.26)	79.91(79.03–80.8)	74.43(73.67–75.19)	<0.0001
Body mass index (kg/m^2^)	27.89(27.7–28.08)	30.28(30.02–30.54)	28.32(27.96–28.68)	27.58(27.3–27.86)	25.5(25.27–25.74)	<0.0001
Systolic blood pressure (mm Hg)	121.35(120.82–121.87)	123.09(122.34–123.83)	121.63(120.68–122.59)	121.55(120.47–122.62)	119.46(118.75–120.17)	<0.0001
Diastolic blood pressure, (mm Hg)	70.87(70.39–71.34)	71.15(70.54–71.76)	70.82(70.07–71.57)	70.88(69.94–71.81)	70.62(70.08–71.17)	<0.0001
Smoking, n (%)	4783(46.31)	1710(46.17)	869(46.17)	636(43.22)	1568(47.88)	0.1003
CKD^a^, n (%)	638(4.35)	326(6.33)	117(4.2)	69(3.68)	126(2.83)	<0.0001
Cardiovascular disease, n (%)	310(2.38)	168(3.53)	46(2.32)	42(2.84)	54(1.1)	<0.0001
Diabetes, n (%)	1555(10.88)	743(15.04)	277(10.87)	208(9.98)	327(7.3)	<0.0001
Total cholesterol, mg/dl	197.03(195.86–198.19)	198.67(196.78–200.55)	199(196.88–201.13)	196.95(193.97–199.93)	194.41(192.81–196.01)	<0.0001
HDL cholesterol, mg/dl	53.26(52.74–53.78)	52.12(51.43–52.81)	52.36(51.34–53.38)	52.78(51.45–54.1)	55.05(54.47–55.64)	<0.0001
Triglycerides, mg/dl	152.94(149.38–156.51)	155.34(151.43–159.26)	163.81(156.57–171.05)	160.12(151.43–168.81)	141.57(136.08–147.06)	<0.0001
Fasting plasma glucose, mg/dl	96.83(96.1–97.56)	99.36(98.18–100.54)	97.26(95.6–98.92)	96.55(95.02–98.08)	94.31(93.16–95.46)	<0.0001
HbA1c^b^, %	5.49(5.46–5.52)	5.6(5.56–5.63)	5.54(5.49–5.58)	5.46(5.42–5.5)	5.39(5.35–5.43)	<0.0001
Blood total clacium, mg/dl	9.46(9.44–9.49)	9.44(9.41–9.47)	9.45(9.42–9.48)	9.47(9.43–9.51)	9.49(9.47–9.51)	<0.0001
Blood phosphorus, mg/dl	3.78(3.77–3.8)	3.75(3.73–3.77)	3.79(3.75–3.83)	3.81(3.78–3.84)	3.8(3.78–3.82)	<0.0001
Vitamin D level, nmol/L	65.4(63.99–66.81)	61.97(60.51–63.43)	65.03(62.81–67.26)	66.46(64.79–68.12)	68.48(66.77–70.18)	<0.0001
eGFR^c^ (MDRD), mL/min/1.73 m^2^	96.69(95.64–97.74)	94.31(93.04–95.59)	95.5(94.08–96.91)	96.12(94.61–97.64)	99.87(98.72–101.03)	<0.0001
eGFR^c^(CKD-EPI), mL/min/1.73 m^2^	95.23(94.3–96.16)	92.41(91.32–93.5)	94.17(92.81–95.53)	94.95(93.5–96.4)	98.63(97.55–99.72)	<0.0001
Protein intake per day, g/kg	1.1(1.09–1.12)	0.58(0.57–0.58)	0.9(0.9–0.9)	1.1(1.09–1.1)	1.72(1.7–1.74)	<0.0001
Calcium intake, mg per day	987.31(964.91–1009.71)	649.51(634.3–664.71)	888.25(860.35–916.16)	1002.37(971.88–1032.85)	1357.93(1321.27–1394.6)	<0.0001
Phosphorus intake, mg per day	1405.18(1384.24–1426.13)	874.22(856.74–891.7)	1242.16(1220.28–1264.03)	1446.45(1422.12–1470.78	1984.07(1952.59–2015.55)	<0.0001
Vitamin D (D2 + D3) intake, mg per day	4.77(4.57–4.97)	2.85(2.7–3.01)	3.91(3.68–4.14)	4.7(4.43–4.96)	7.14(6.62–7.66)	<0.0001
Calorie intake, kcal/day/kg	28.14(27.74–28.53)	17.87(17.58–18.16)	24.7(24.27–25.13)	28.59(28.2–28.97)	39.63(38.94–40.32)	<0.0001
Calorie intake, kcal/day	2174.91(2145.87–2203.95)	1493.59(1462.34–1524.84)	1978.7(1936.71–2020.68)	2259.86(2216.25–2303.47)	2896.68(2847.67–2945.69)	<0.0001
% from carbohydrate	49.74(49.34–50.13)	53.83(53.33–54.32)	49.94(49.3–50.59)	49.07(48.5–49.64)	46.01(45.53–46.49)	<0.0001
% from fat	34.2(33.9–34.51)	32.4(31.98–32.83)	34.4(33.82–34.98)	34.56(34.05–35.07)	35.65(35.24–36.07)	<0.0001
% from protein	16.06(15.9–16.22)	13.77(13.56–13.98)	15.66(15.4–15.92)	16.37(16.12–16.62)	18.34(18.07–18.6)	<0.0001
L1 BMD^d^	0.957(0.953–0.961)	0.96(0.952–0.968)	0.96(0.952–0.968)	0.956(0.947–0.965)	0.953(0.947–0.958)	<0.0001
L1 T score	−2.024(-2.061–1.987)	−1.958(-2.022–1.895)	−1.989(-2.051–1.926)	−2.041(-2.12–1.962)	−2.098(-2.141–2.055)	<0.0001
L2 BMD^d^	1.047(1.043–1.051)	1.051(1.044–1.059)	1.054(1.048–1.061)	1.045(1.034–1.055)	1.04(1.034–1.047)	<0.0001
L2 T score	−1.272(-1.309–1.236)	−1.199(-1.258–1.14)	−1.201(-1.255–1.147)	−1.303(-1.39–1.216)	−1.368(-1.421–1.315)	<0.0001
L3 BMD^d^	1.07(1.065–1.074)	1.078(1.071–1.085)	1.08(1.073–1.088)	1.063(1.053–1.072)	1.059(1.052–1.066)	<0.0001
L3 T score	−1.085(-1.122–1.047)	−0.977(-1.036–0.918)	−0.987(-1.049–0.925)	−1.152(-1.235–1.07)	−1.211(-1.267–1.156)	<0.0001
L4 BMD^d^	1.068(1.064–1.073)	1.078(1.071–1.085)	1.078(1.071–1.086)	1.064(1.055–1.073)	1.056(1.049–1.063)	<0.0001
L4 T score	−1.094(-1.129–1.058)	−0.976(-1.034–0.918)	−1.001(-1.065–0.936)	−1.144(-1.222–1.067)	−1.236(-1.293–1.178)	<0.0001
All BMD^d^	1.04(1.035–1.044)	1.046(1.039–1.053)	1.047(1.041–1.054)	1.036(1.027–1.045)	1.031(1.025–1.036)	<0.0001
All T score	−1.335(-1.369–1.301)	−1.242(-1.298–1.186)	−1.259(-1.315–1.204)	−1.378(-1.456–1.3)	−1.448(-1.498–1.398)	<0.0001

^a^ CKD, chronic kidney disease.

^b^ HbA1c, glycated hemoglobin.

^c^ eGFR, estimated glomerular filtration rate.

^d^ BMD, bone mineral density. Continuous variable: mean (95% CI).

### Lumbar BMD and T score divided by different protein intake

We investigated BMD and T-scores from different protein diets after adjusting for age, sex, daily energy intake, BW, and CKD (Table 2). After adjustments, the above variables showed no significant difference in bone density (both BMD and T score) with respect to different protein intake. The above findings were further divided into two groups: CKD and non-CKD (Table 3, MDRD formula). Again, we found no significant differences among all the comparisons. Nevertheless, there was an apparent trend showing that in the CKD group, BMD decreased with increasing DPI in all single lumbar spines (L1, L2, L3, and L4) and all L spines (L1-L4) (Supplementary Figure 1B). This trend was not observed in the non-CKD group (Supplementary Figure 1A). Similarly, this trend was also observed in the CKD group, as with the decreasing T score with increasing DPI at all single lumbar spines (L1, L2, L3, and L4) and all L spines (L1-L4) (Supplementary Figure 1D). This trend was not observed in the non-CKD group (Supplementary Figure 1C). The above conditions were also similar when CKD was defined using the CKD-EPI formula (Supplementary Table 1).

**Table 2.** Lumbar BMD and T score according to different level of daily protein intake.

Region of interest		Daily protein intake per day, g/kg				*p* for trend
		<0.8	0.8–1.0	1.0–1.2	>1.2	
L1	BMD	0.955 ± 0.012	0.961 ± 0.011	0.956 ± 0.013	0.957 ± 0.011	0.8465
	T SCORE	−2.038 ± 0.041	−1.991 ± 0.049	−2.03 ± 0.061	−2.021 ± 0.051	0.8465
L2	BMD	1.046 ± 0.01	1.056 ± 0.009	1.047 ± 0.011	1.049 ± 0.009	0.8098
	T SCORE	−1.288 ± 0.032	−1.197 ± 0.04	−1.273 ± 0.056	−1.26 ± 0.052	0.8098
L3	BMD	1.071 ± 0.011	1.083 ± 0.01	1.068 ± 0.012	1.073 ± 0.011	0.8411
	T SCORE	−1.075 ± 0.042	−0.976 ± 0.049	−1.099 ± 0.059	−1.058 ± 0.06	0.8411
L4	BMD	1.069 ± 0.011	1.079 ± 0.009	1.067 ± 0.01	1.07 ± 0.01	0.9114
	T SCORE	−1.096 ± 0.031	−1.01 ± 0.043	−1.107 ± 0.044	−1.08 ± 0.053	0.9114
All	BMD	1.039 ± 0.01	1.049 ± 0.008	1.039 ± 0.01	1.041 ± 0.009	0.9914
	T SCORE	−1.34 ± 0.035	−1.259 ± 0.044	−1.344 ± 0.053	−1.321 ± 0.052	0.9914

Adjustments for age, sex, daily energy intake, body weight, and CKD.

**Table 3.** Lumbar BMD and T score according to different level of daily protein intake divided by CKD or not (MDRD-eGFR).

Region of interest	CKD or not (MDRD)	Daily protein intake per day, g/kg				P for trend	*o* for interaction
		<0.8	0.8–1.0	1.0–1.2	>1.2		
BMD							
L1	Non CKD	0.952 ± 0.004	0.959 ± 0.004	0.954 ± 0.005	0.955 ± 0.003	0.7808	0.9200
	CKD	0.93 ± 0.01	0.914 ± 0.015	0.913 ± 0.021	0.907 ± 0.017	0.3176	
L2	Non CKD	1.041 ± 0.004	1.052 ± 0.004	1.043 ± 0.005	1.045 ± 0.004	0.7274	0.5980
	CKD	1.022 ± 0.011	1.01 ± 0.014	1.002 ± 0.025	0.997 ± 0.019	0.2708	
L3	Non CKD	1.063 ± 0.004	1.077 ± 0.004	1.061 ± 0.005	1.067 ± 0.004	0.9568	0.6264
	CKD	1.071 ± 0.012	1.036 ± 0.015	1.042 ± 0.026	1.027 ± 0.017	0.0683	
L4	Non CKD	1.063 ± 0.004	1.075 ± 0.004	1.062 ± 0.004	1.066 ± 0.004	0.9531	0.7801
	CKD	1.077 ± 0.01	1.056 ± 0.014	1.055 ± 0.028	1.047 ± 0.018	0.2233	
All	Non CKD	1.034 ± 0.004	1.045 ± 0.004	1.034 ± 0.005	1.037 ± 0.003	0.8619	0.9952
	CKD	1.03 ± 0.01	1.009 ± 0.013	1.009 ± 0.025	0.999 ± 0.017	0.1716	
T score							
L1	Non CKD	−2.065 ± 0.036	−2.011 ± 0.032	−2.054 ± 0.038	−2.043 ± 0.022	0.7808	0.9200
	CKD	−2.25 ± 0.085	−2.384 ± 0.124	−2.391 ± 0.175	−2.439 ± 0.14	0.3176	
L2	Non CKD	−1.328 ± 0.033	−1.232 ± 0.031	−1.31 ± 0.042	−1.294 ± 0.03	0.7274	0.5980
	CKD	−1.483 ± 0.091	−1.586 ± 0.118	−1.652 ± 0.21	−1.692 ± 0.155	0.2708	
L3	Non CKD	−1.14 ± 0.035	−1.025 ± 0.034	−1.156 ± 0.04	−1.107 ± 0.032	0.9568	0.6264
	CKD	−1.078 ± 0.099	−1.364 ± 0.126	−1.319 ± 0.216	−1.442 ± 0.142	0.0683	
L4	Non CKD	−1.141 ± 0.035	−1.045 ± 0.036	−1.148 ± 0.037	−1.115 ± 0.034	0.9531	0.7801
	CKD	−1.028 ± 0.085	−1.2 ± 0.115	−1.211 ± 0.236	−1.271 ± 0.149	0.2233	
All	Non CKD	−1.385 ± 0.033	−1.294 ± 0.031	−1.385 ± 0.038	−1.357 ± 0.028	0.8619	0.9952
	CKD	−1.42 ± 0.082	−1.591 ± 0.111	−1.589 ± 0.204	−1.673 ± 0.138	0.1716	

Adjustments for age, sex, daily energy intake, body weight, and CKD. CKD: chronic kidney disease.

### Relative risk of osteoporosis in lumbar spines divided by different protein intake

The outcome of osteoporosis in lumbar spines is shown in Table 4 (MDRD formula for CKD definition) against different levels of DPI (as compared with 0.8–1.0 g per day/kg of DPI). After adjustment for age, gender, daily energy intake and BW, compared with DPI (0.8–1.0 g/day/kg), higher risks of osteoporosis were noticed in subjects with intakes >1.2 g/day/kg over L2 (RR = 1.326, 95%CI = 1.062–1.656), >1.2 g/day/kg over L3 (RR = 1.31, 95%CI = 1.057–1.622), <0.8 g/day/kg over L4 (RR = 1.276, 95%CI = 1.015–1.605), <0.8 g/day/kg over all L spines (RR = 11.275, 95%CI = 1.051–1.548), and >1.2 g/day/kg over all L spines (RR = 1.333, 95%CI = 1.098–1.618). Further segregation into CKD and non-CKD groups, higher risks of osteoporosis were only found in non-CKD group (supplementary Figure 1E): <0.8 g/day/kg over L2 (RR = 1.352, 95%CI = 1.104–1.656), >1.2 g/day/kg (RR = 1.315, 95%CI = 1.037–1.668), >1.2 g/day/kg (RR = 1.322, 95%CI = 1.058–1.651), <0.8 g/day/kg over all L spine (RR = 1.281, 95%CI = 1.038–1.581), and >1.2 g/day/kg (RR = 1.357, 95%CI = 1.114–1.654). In summary, in the non-CKD group, DPI levels of too low (<8 g/day/kg) and too high (>1.2 g/day/kg) were associated with higher risks of osteoporosis over L2 and all L spines, mimicking a U-shaped function (Supplementary Figure 1E). In the CKD group, the risk of osteoporosis was not associated with the DPI (Supplementary Figure 1F). The above condition can also be observed in the CKD-EPI formula-based CKD definition (Supplementary Table 2).

**Table 4.** Relative risk of osteoporosis in lumbar spines in different level of daily protein diet (compared to 0.8–1.0 g/day/kg of daily protein intake) divided by CKD or not (MDRD-eGFR).

Region of interest	Daily protein intake per day, g/kg				P for trend	*p* for interaction
	<0.8	0.8–1.0	1.0–1.2	>1.2		
L1	0.985(0.849–1.142)	REF	0.941(0.767–1.154)	0.957(0.807–1.135)	0.6712	
Non-CKD^a^	0.978(0.838–1.142)	REF	0.941(0.764–1.159)	0.95(0.796–1.135)	0.6807	0.5079
CKD	1.008(0.597–1.701)	REF	0.996(0.449–2.207)	1.138(0.627–2.068)	0.7789	
L2	1.352(1.128–1.621)	REF	1.163(0.912–1.484)	1.326(1.062–1.656)	0.9165	
Non-CKD	1.352(1.104–1.656)	REF	1.154(0.896–1.486)	1.315(1.037–1.668)	0.9624	0.2183
CKD	1.115(0.605–2.055)	REF	1.528(0.669–3.49)	1.45(0.828–2.54)	0.3861	
L3	1.222(0.96–1.556)	REF	1.099(0.877–1.377)	1.31(1.057–1.622)	0.4315	
Non-CKD	1.22(0.949–1.569)	REF	1.114(0.88–1.409)	1.322(1.058–1.651)	0.4118	0.4010
CKD	1.179(0.608–2.287)	REF	0.99(0.303–3.229)	1.138(0.484–2.672)	0.9150	
L4	1.276(1.015–1.605)	REF	1.13(0.865–1.477)	1.221(0.921–1.619)	0.9241	
Non-CKD	1.269(0.994–1.62)	REF	1.144(0.86–1.522)	1.23(0.924–1.637)	0.9872	0.4190
CKD	1.283(0.649–2.533)	REF	1.019(0.325–3.189)	1.059(0.471–2.381)	0.7295	
All	1.275(1.051–1.548)	REF	1.169(0.902–1.515)	1.333(1.098–1.618)	0.4951	
Non-CKD	1.281(1.038–1.581)	REF	1.196(0.919–1.557)	1.357(1.114–1.654)	0.4286	0.1763
CKD	1.068(0.553–2.062)	REF	0.941(0.326–2.716)	1.011(0.45–2.271)	0.8858	

Adjustment for age, sex, daily energy intake, and body weight.

^a^ CKD: chronic kidney disease (MDRD formula).

### Relative risk of osteoporosis divided by different protein intake and elderly or not

The relative risk of osteoporosis according to age is shown in Supplementary Table 3, and according to CKD is shown in Table 2. A higher relative risk of osteoporosis was found in the elderly and low protein intake (LPI) (<0.8 g/day/kg) over L2 (RR = 1.399, 92% CI = 1.03–1.9), L3 (RR = 1.523, 95% CI = 1.015–2.286) and all L spines (RR = 1.479, 95% CI = 1.2–2.186) (Supplementary Table 3). Further taking CKD or not into consideration (supplementary Table 4), non-CKD and the elderly posed higher risks for osteoporosis, including LPI (<0.8 g/day/kg) for L2 (RR = 1.483, 95%CI = 1.002–2.195), LPI (<0.8 g/day/kg) for L3 (RR = 1.73, 95%CI = 1.045–2.864), LPI (<0.8 g/day/kg) for all L spines (RR = 1.673, 95%CI = 1.02–2.647), and HPI (1.0–1.2 g/day/kg) for all L spines (RR = 1.579, 95%CI = 1.033–2.414).

### Relative risk of osteoporosis divided by different protein intake, calcium, phosphate, and vitamin D

The relative risk for L spine osteoporosis among different daily calcium and phosphorus intakes and blood calcium and phosphorus intakes are summarized in supplementary Tables 5–10. As shown in supplementary Table 9 (calcium and phosphorus intake), low calcium and low phosphorus intake were associated higher risks for osteoporosis in LPI (<0.8 g/day/kg) over L2 (RR = 1.367, 95%CI = 1.068–1.749) and high DPI (>1.2 g/day/kg) over L2 (RR = 1.971, 95%CI = 1.378–2.818), L3 (RR = 1.641, 95%CI = 1.099–2.451), L4 (RR = 1.645, 95%CI = 1.111–2.436), and all L spines (RR = 1.883, 95%CI = 1.324–2.677). High calcium and phosphorus intakes were associated with higher risks for osteoporosis LPI (<0.8/day/kg) over L2 (RR = 1.619, 95%CI = 1.055–2.485) and all L spines (RR = 1.725, 95%CI = 1.09–2.729). In supplementary Table 10 (blood calcium and phosphorus levels), low blood calcium and low blood phosphorus levels were associated higher risks for osteoporosis in LPI (<0.8 g/day/kg) over L2 (RR = 1.8, 95%CI = 1.234–2.627), L3 (RR = 1.724, 95%CI = 1.094–2.716), L4 (RR = 2.247, 95%CI = 1.412–3.574) and all L spines (RR = 1.853, 95%CI = 1.212–2.834); HPI (1.0–1.2 g/day/kg) over L4 (RR = 2.5, 95%CI = 1.39–4.497) and all L spines (RR + 2.074, 95%CI = 1.195–3.598); VHPI (>1.2 g/day/kg) over L2 (RR = 1.726, 95%CI = 1.102–2.703), L4 (RR = 1.892, 95%CI = 1.097–3.262) and all L spines (RR = 1.777, 95%CI = 1.104–2.860). High blood calcium and high blood phosphorus levels were associated with higher risk for osteoporosis in LPI (<0.8 g/day/kg) over L2 (RR = 1.399, 95%CI = 1.028–1.905) and all L spines (RR = 1.547, 95%CI = 1.081–2.215) and VHPI (>1.2 g/day/kg) over all L spines (RR = 1.485, 95%CI = 1.066–2.069). In summary, only calcium and phosphorus levels (intake or blood value) that were too low or too high were associated with a higher risk of osteoporosis.

The associations between daily vitamin D intake and blood vitamin D concentrations are shown in Supplementary Tables 11 and 12. High vitamin D intake and LPI (<0.8 g/day/kg) were associated with a higher risk of osteoporosis in L2 (RR = 1.785, 955CI = 1.289–2.473), L4 (RR = 1.42, 95%CI = 1.018–1.979) and all L spines (RR = 1.394, 95%CI = 1.026–1.894). High vitamin D blood concentration and LPI (<0.8 g/day/kg) were associated with higher risks of osteoporosis in L2 (RR = 1.729, 95%CI = 1.312–2.278) and all L spines (RR = 1.558, 95%CI = 1.164–2.086).

## Discussion

In our previous study on femoral BMD in the same cohort regarding femoral BMD (supplementary Tables 13–15) [31], we found that HPI is associated with higher femoral BMD in patients without CKD. CKD patients with HPI do not benefit from developing higher femoral BMD, and those with LDI do not develop lower femoral BMD. However, in a previously published study [55], it was found that a high dietary inflammation index is independently associated with decreased bone mineral density (BMD) in the femoral regions. In the present study, we found similar results regarding lumbar BMD in the NHANES cohort. As shown in Table 1, patients with a higher DPI experienced lower BMD and T scores. This association disappeared after adjusting for age, sex, daily energy intake, BW and CKD (Table 2). Furthermore, the different DPI groups were not associated with BMD and T scores in either the CKD or non-CKD patient groups (Table 3). When viewing such an association in diagrams (Supplementary Figure 1), we observed an apparent trend that higher DPI was associated with lower BMD, despite having no statistical significance (Supplementary Figure 1A) and T score (Supplementary Figure 1C), despite having no statistical significance. As for osteoporosis (Table 4) (reference:0.8–1.0 g/day/kg), lower (<0.8 g/day/kg) or higher DPI (>1.2 g/day/kg) was associated with higher risks for osteoporosis in non-CKD group, but not in the CKD group. In summary, in the CKD group (defined by either MDRD or CKD-EPI), our nephrologists considered that the LPI for renal protection was safe without threatening L spine bone density (BMD and T score) and without higher risks for osteoporosis. LPI might also have potential benefits on L-spine bone health (trend with higher BMD and T score).

Currently, in the general adult population (≥18 y/o), the RDA for protein intake is 0.8 g/kg/day [4], which has remained unchanged over the past 70 years. Adequate protein intake is essential for continuous turnover and remodeling because proteins make up 50% of the bone and 1/3 of the bone mass. Proteins are detrimental and beneficial to bone health [56]. Therefore, the ideal DPI for osteoporosis remains debatable. Recently, the International Osteoporosis Foundation, National Osteoporosis Foundation, American Bone Health, and American Society for Nutrition have understood the role of DPI in optimizing bone health throughout the lifespan [57]. Four systematic reviews suggested that high DPI has no detrimental effect on bone health and may benefit elderly populations [58–60], But the studies on the ideal amount of DPI for osteoporosis in the CKD population are still limited. Our data (Supplementary Tables 3 and 4) showed an association between different DPI and age in the non-CKD group, but not in the CKD group.

The influence of DPI on bone health may depend on calcium intake. A high DPI may have desirable effects on changes in hip BMD in the elderly population when supplemented with calcium citrate malate and vitamin D [61]. Another study in middle-aged people reported that high DPI coupled with calcium intake (800 mg/day or more) could protect against hip fracture [62]. BMD may be improved by increasing the DPI only when meeting the recommended intake of calcium and vitamin D. Our data (Supplementary Tables 5, 6, 11, and 12) also indicated that a mismatch between DPI and daily calcium or vitamin D intake was associated with a higher risk of osteoporosis in L spine in the non-CKD group. This condition also echoed DPI’s non-apparent effect of DPI on bone density in the CKD population when daily calcium and vitamin D intakes were not matched. According to the KDIGO guideline [30], calcium and vitamin D supplements in patients with CKD are not as simple as those in the non-CKD population. In the CKD population, hyperphosphatemia and secondary hyperparathyroidism arise if the eGFR drops below 60 mL/min/1.732 m^2^ [63]. The initial treatment is to deal with hyperphosphatemia by restricting phosphate intake and by taking calcium-based phosphate binders (calcium carbonate and calcium acetate) in meals to lower the excess phosphate [64–66]. This intake of calcium-containing phosphate binders does not elevate blood calcium levels. Calcium intake before or after meals is a contraindication for CKD patients to avoid extraskeletal calcium phosphate deposition [67–69], particularly during hyperphosphatemia. Similar to calcium supplementation, vitamin D supplementation in CKD patients is indicated to control elevated parathyroid levels instead of osteoporosis. Therefore, mismatched Ca-vitamin D and DPI levels are common among patients with CKD. This mismatch may explain the minimal association between DPI and bone density in the CKD population.

Other issues need to be addressed regarding LPI in patients with CKD. First, insufficient protein intake may lead to a shortage of materials for bone turnover and metabolism. That’s why hypoalbuminemia differently affects the serum bone turnover markers in hemodialysis patients and serum albumin measurement should be considered according to previous study [70]. However, increased acidosis (particularly from animal origin with sulfur-containing amino acids) is deleterious to the skeleton, leading to osteoporosis and an enhanced risk of fragility fracture [5]. A lower DPI leads to less acidosis, which is beneficial to bone density. In patients with CKD, metabolic acidosis is almost always due to the production of sulfuric acid from metabolizing sulfur-containing amino acids [71,72]. Chronic metabolic acidosis in chronic kidney disease (CKD) can cause bone resorption and osteoporosis [73–75]. Therefore, a low DPI is beneficial for bone density as far as acidosis is concerned. Third, LPD is the standard treatment for CKD to gain better renal function, followed by fewer CKD-mineral bone diseases. Hence, LPI in CKD is associated with less CKD-mineral bone disease owing to the improvement of bone metabolism and insulin sensitivity [75]. In summary, the effect of different DPI levels on bone density is a complex process in patients with CKD. Here, we present the first study with evidence to conclude that the association between different DPI and bone density is not found in CKD patients.

This study has some limitations. First, the benefits of physical activity for the management or prevention of osteoporosis have been published [76]. This association was also confirmed in the cohort of NHANES [77]. However, greater physical activity has been reported to be associated with more protein intake [78]. Therefore, even without data on physical activity, the association between bone density and protein intake can still be investigated. Second, we did not have data on pharmacological anti-osteoporosis therapies in this cohort. We only collected data on calcium and vitamin D supplements because calcium and vitamin D are both non-pharmacological or nutritional interventions. The aim of this study was nutritional intervention (different protein intakes). We only collected data associated with nutritional interventions, including calcium, vitamin D, and protein intake. In addition, in our previous study [31], the lack of benefit of higher protein intake on bone health of the hip joint may be due to insufficient calcium and vitamin D supplementation. Therefore, calcium and vitamin D interventions provided much more important information in this study. Third, we acknowledge that other mineral materials might affect bone health. Unfortunately, we lacked data on other serum mineral materials within the NHANES database. Fourth, in this cohort, renal function remains relatively robust, and the CKD population is still in the early stages. Therefore, the relationship between various protein diets and bone health in the later stages of CKD cannot be assessed within the scope of the current study. Finally, this is a retrospective study, and there may still be some unidentified confounding factors.

## Conclusion

Based on the NHANES data, different amounts of DPI were not significantly associated with BMD and T scores in both the CKD and non-CKD groups (either MDRD or CKD-EPI formula). However, there was an apparent trend in patients with CKD, despite no statistical significance, that higher DPI was associated with lower BMD and T scores. As for osteoporosis (reference:0.8–1.0 g/day/kg), lower (<0.8 g/day/kg) or higher DPI (>1.2 g/day/kg) was associated with higher risks for osteoporosis in non-CKD group, but not in the CKD group. In summary, in the CKD group, LPI provided safe renal protection without threatening L spine bone density (BMD and T score) and without increasing the risk of osteoporosis.

## Supplementary Material

Supplemental Material

## Data Availability

The datasets used and/or analyzed during the current study are available from the corresponding author upon reasonable request.
