# Supplementary material for: The association between bone density of lumbar spines and different daily protein intake in different renal function
Source: Ren Fail. 2024 Jan 8;46(1):2298080. doi: 10.1080/0886022X.2023.2298080 (PMC10776072; doi:10.1080/0886022X.2023.2298080)
Supplement: Supplemental Material [file IRNF_A_2298080_SM2766.pdf]

Supplementary data

Table 1. Lumbar BMD and T score according to different level of daily protein intake divided by CKD or not (CKD-EPI formula).

Region of interest		Protein				P for trend	P for interaction
		<0.8	0.8-1.0	1.0-1.2	>1.2		
				**BMD**			
L1	Non CKD	0.952±0.004	0.959±0.004	0.954±0.005	0.955±0.003	0.7653	0.8822
	CKD	0.934±0.01	0.92±0.014	0.916±0.02	0.91±0.016	0.2530	
L2	Non CKD	1.041±0.004	1.052±0.004	1.043±0.005	1.045±0.004	0.7046	0.7807
	CKD	1.028±0.011	1.017±0.013	1.005±0.023	0.999±0.018	0.1889	
L3	Non CKD	1.063±0.004	1.077±0.004	1.061±0.005	1.067±0.004	0.9494	0.4790
	CKD	1.074±0.012	1.042±0.014	1.046±0.024	1.03±0.017	0.0577	
L4	Non CKD	1.063±0.004	1.074±0.004	1.062±0.004	1.066±0.004	0.9347	0.5661
	CKD	1.082±0.01	1.061±0.013	1.058±0.026	1.049±0.018	0.1542	0.7767
All	Non CKD	1.034±0.004	1.045±0.004	1.034±0.005	1.037±0.003	0.8442	
	CKD	1.034±0.01	1.015±0.012	1.013±0.023	1.002±0.017	0.1252	
				**T score**			
L1	Non CKD	-2.064±0.036	-2.01±0.033	-2.054±0.039	-2.042±0.022	0.7653	0.8822
	CKD	-2.215±0.08	-2.335±0.117	-2.364±0.167	-2.418±0.137	0.2530	
L2	Non CKD	-1.328±0.034	-1.232±0.031	-1.31±0.042	-1.293±0.03	0.7046	0.7807
	CKD	-1.434±0.088	-1.525±0.11	-1.626±0.193	-1.676±0.154	0.1889	
L3	Non CKD	-1.14±0.035	-1.024±0.034	-1.157±0.04	-1.107±0.032	0.9494	0.4790
	CKD	-1.048±0.096	-1.32±0.117	-1.284±0.199	-1.419±0.141	0.0577	
L4	Non CKD	-1.144±0.035	-1.046±0.036	-1.149±0.037	-1.116±0.034	0.9347	0.5661
	CKD	-0.985±0.083	-1.156±0.106	-1.184±0.219	-1.258±0.147	0.1542	0.7767
All	Non CKD	-1.386±0.033	-1.294±0.032	-1.385±0.038	-1.357±0.028	0.8442	
	CKD	-1.38±0.081	-1.542±0.102	-1.56±0.19	-1.654±0.138	0.1252	

Adjust for age, gender, energy, weight

Information Classification: General

Table 2. Relative risk of osteoporosis in lumbar spines in different level of daily protein diet (compared to 0.8-1.0 g/day/kg of daily protein intake) divided by CKD or not (CKD-EPI formula).

Region of interest		Protein intake per day/kg				P for trend	P for interaction
		<0.8	0.8-1.0	1.0-1.2	>1.2		
L1	Non-CKD[#]	0.979(0.837-1.144)	REF	0.943(0.764-1.163)	0.951(0.797-1.137)	0.6837	0.5896
	CKD	1.007(0.6-1.69)	REF	0.976(0.455-2.091)	1.108(0.593-2.069)	0.8291	
L2	Non-CKD	1.356(1.106-1.662)	REF	1.159(0.899-1.493)	1.318(1.039-1.673)	0.9624	0.3305
	CKD	1.136(0.641-2.013)	REF	1.435(0.655-3.142)	1.455(0.816-2.593)	0.4349	
L3	Non-CKD	1.23(0.956-1.583)	REF	1.123(0.887-1.422)	1.322(1.057-1.653)	0.4433	0.6852
	CKD	1.13(0.603-2.118)	REF	0.88(0.272-2.845)	1.203(0.518-2.797)	0.9626	
L4	Non-CKD	1.279(1-1.636)	REF	1.152(0.866-1.533)	1.235(0.928-1.643)	0.9984	0.4641
	CKD	1.163(0.589-2.298)	REF	0.946(0.316-2.833)	1.06(0.468-2.401)	0.8457	
ALL	Non-CKD	1.287(1.042-1.589)	REF	1.203(0.924-1.566)	1.359(1.114-1.659)	0.4396	0.3120
	CKD	1.085(0.585-2.012)	REF	0.878(0.311-2.477)	1.034(0.47-2.273)	0.8646	

Adjustment for age, gender, daily energy intake and body weight. [#]CKD: chronic kidney disease (CKD-EPI formula)

Information Classification: General

Table 3. Relative risk of osteoporosis in lumbar spines in different level of protein diet (compared to 0.8-1.0 g per day/kg of daily protein intake) subgrouped by the **elderly or not.**

Region of interest	Age	Daily protein intake per day, g/kg				P for trend	P for interaction
		<0.8	0.8-1.0	1.0-1.2	>1.2		
L1	<65	0.948(0.798-1.127)	REF	0.933(0.77-1.129)	0.938(0.787-1.117)	0.7922	0.5707
	≥65	1.039(0.749-1.443)	REF	1.056(0.665-1.677)	0.852(0.526-1.38)	0.5628	
L2	<65	1.263(0.995-1.602)	REF	1.122(0.853-1.474)	1.245(0.947-1.636)	0.8809	0.6883
	≥65	1.399(1.03-1.9)	REF	1.406(0.926-2.134)	1.238(0.768-1.995)	0.8335	
L3	<65	1.13(0.857-1.491)	REF	1.055(0.807-1.378)	1.246(0.949-1.635)	0.4183	0.8174
	≥65	1.523(1.015-2.286)	REF	1.281(0.825-1.988)	1.063(0.657-1.721)	0.2662	
L4	<65	1.207(0.938-1.552)	REF	1.095(0.787-1.524)	1.194(0.86-1.657)	0.9117	0.4877
	≥65	1.482(0.958-2.29)	REF	1.285(0.809-2.039)	0.881(0.494-1.569)	0.1109	
All	<65	1.184(0.935-1.499)	REF	1.114(0.827-1.501)	1.279(1.01-1.62)	0.4136	0.9562
	≥65	1.479(1-2.186)	REF	1.451(0.976-2.156)	1.125(0.729-1.734)	0.5004	

Adjustment for gender, daily energy intake and body weight

Information Classification: General

Table 4. Relative risk of osteoporosis in lumbar spines in different level of protein diet (compared to 0.8-1.0 g per day/kg of daily protein intake) subgrouped by the **CKD and the elderly or not.**

Region of interest	Ange and CKD	Daily protein intake per day, g/kg				P for trend	P for interaction
		<0.8	0.8-1.0	1.0-1.2	>1.2		
L1	Age<65,Non-CKD	0.98(0.823-1.168)	REF	0.929(0.755-1.143)	0.947(0.787-1.141)	0.6328	0.9848
	Age <65,CKD	0.884(0.232-3.366)	REF	0.915(0.224-3.74)	3.049(0.705-13.189)	0.1661	
	Age ≥65,Non-CKD	1.032(0.687-1.548)	REF	1.081(0.655-1.785)	0.9(0.501-1.618)	0.7650	
	Age ≥65, CKD	1.055(0.59-1.884)	REF	1.017(0.38-2.723)	0.759(0.352-1.636)	0.4926	
L2	Age <65, non-CKD	1.347(1.059-1.714)	REF	1.114(0.83-1.496)	1.301(0.982-1.724)	0.9851	0.1556
	Age <65, CKD	1.41(0.291-6.818)	REF	2.185(0.348-13.715)	1.899(0.378-9.549)	0.6641	
	Age ≥65, non-CKD	1.483(1.002-2.195)	REF	1.425(0.928-2.187)	1.253(0.74-2.124)	0.7348	
	Age >=65, CKD	1.091(0.55-2.166)	REF	1.406(0.523-3.778)	1.356(0.721-2.55)	0.5536	
L3	Age <65, non-CKD	1.18(0.896-1.555)	REF	1.067(0.801-1.421)	1.296(0.988-1.7)	0.4227	0.6143
	Age <65, CKD	2.988(0.55-16.216)	REF	0.329(0.026-4.224)	5.156(0.657-40.445)	0.7212	
	Age ≥65, non-CKD	1.73(1.045-2.864)	REF	1.366(0.81-2.303)	1.265(0.72-2.224)	0.3605	
	Age ≥65, CKD	0.989(0.454-2.152)	REF	1.099(0.343-3.52)	0.711(0.274-1.85)	0.6404	
L4	Age <65, non-CKD	1.26(0.967-1.642)	REF	1.124(0.794-1.591)	1.241(0.891-1.727)	0.8998	0.2110
	Age <65, CKD	1.422(0.344-5.874)	REF	0.138(0.013-1.432)	1.682(0.331-8.559)	0.9801	
	Age ≥65, non-CKD	1.533(0.916-2.566)	REF	1.278(0.763-2.141)	0.944(0.457-1.954)	0.1947	
	Age ≥65, CKD	1.291(0.53-3.142)	REF	1.368(0.427-4.386)	0.774(0.308-1.947)	0.5316	
All	Age <65, non-CKD	1.249(0.98-1.592)	REF	1.137(0.825-1.566)	1.331(1.046-1.693)	0.4489	0.4125
	Age <65, CKD	1.447(0.305-6.868)	REF	0.164(0.016-1.719)	2.61(0.48-14.176)	0.7315	
	Age ≥65, non-CKD	1.643(1.02-2.647)	REF	1.579(1.033-2.414)	1.333(0.803-2.212)	0.6674	
	Age ≥65, CKD	1.009(0.451-2.255)	REF	1.205(0.405-3.581)	0.709(0.264-1.905)	0.6395	

Adjustment for gender, daily energy intake and body weight

Information Classification: General

Table 5. Relative risk of osteoporosis in lumbar spines in different level of protein diet (compared to 0.8-1.0 g per day/kg of daily protein intake) subgrouped by the **daily calcium intake level**

Region of interest	Calcium Intake (856 (mg))	Daily protein intake per day, g/kg				P for trend	P for interaction
		<0.8	0.8-1.0	1.0-1.2	>1.2		
L1	<median	0.961(0.787-1.175)	REF	0.869(0.653-1.157)	0.972(0.755-1.251)	0.8618	0.8961
	≥median	1(0.729-1.372)	REF	1.014(0.772-1.333)	0.968(0.754-1.242)	0.8167	
L2	<median	1.243(0.972-1.588)	REF	1.036(0.793-1.355)	1.543(1.167-2.038)	0.3120	0.4208
	≥median	1.444(1.117-1.867)	REF	1.31(0.898-1.909)	1.221(0.882-1.691)	0.8716	
L3	<median	1.197(0.896-1.6)	REF	0.904(0.649-1.259)	1.439(1.067-1.941)	0.5782	0.3054
	≥median	1.171(0.756-1.814)	REF	1.299(0.877-1.925)	1.259(0.879-1.804)	0.3068	
L4	<median	1.189(0.888-1.591)	REF	0.957(0.656-1.397)	1.451(1.043-2.02)	0.4331	0.7499
	≥median	1.407(0.936-2.115)	REF	1.334(0.884-2.014)	1.13(0.748-1.707)	0.6943	
All	<median	1.199(0.919-1.565)	REF	1.038(0.763-1.413)	1.497(1.12-2.001)	0.3036	0.9647
	≥median	1.447(1.045-2.003)	REF	1.309(0.899-1.904)	1.249(0.92-1.696)	0.9812	

Adjustment for age, gender, daily energy intake and body weight

Information Classification: General

Table 6. Relative risk of osteoporosis in lumbar spines in different level of protein diet (compared to 0.8-1.0 g per day/kg of daily protein intake) subgrouped by the **blood calcium concentration**

Region of interest	Blood calcium, (9.4(mg/dL))	Daily protein intake per day, g/kg				P for trend	P for interaction
		<0.8	0.8-1.0	1.0-1.2	>1.2		
L1	<median	0.947(0.77-1.165)	REF	0.929(0.703-1.228)	0.906(0.728-1.128)	0.6356	0.6850
	≥median	1.031(0.861-1.234)	REF	0.96(0.714-1.291)	1.016(0.816-1.264)	0.8592	
L2	<median	1.374(1.033-1.827)	REF	1.384(1.047-1.828)	1.361(0.997-1.857)	0.6714	0.9617
	≥median	1.331(1.055-1.68)	REF	0.994(0.666-1.483)	1.308(0.978-1.751)	0.9195	
L3	<median	1.39(0.959-2.016)	REF	1.444(0.999-2.086)	1.379(0.951-2.001)	0.6679	0.9234
	≥median	1.1(0.793-1.525)	REF	0.86(0.593-1.246)	1.258(0.907-1.745)	0.5001	
L4	<median	1.371(0.989-1.901)	REF	1.459(0.982-2.166)	1.265(0.812-1.971)	0.9766	0.7198
	≥median	1.197(0.902-1.589)	REF	0.884(0.627-1.247)	1.186(0.844-1.666)	0.9044	
All	<median	1.302(0.961-1.765)	REF	1.503(1.091-2.07)	1.293(0.956-1.748)	0.5810	0.5328
	≥median	1.251(0.992-1.578)	REF	0.909(0.611-1.352)	1.373(1.007-1.872)	0.5916	

Adjust for age, gender, daily energy intake and body weight

Information Classification: General

Table 7. Relative risk of osteoporosis in lumbar spines in different level of protein diet (compared to 0.8-1.0 g per day/kg of daily protein intake) subgrouped by the **daily phosphorus intake level**

Region of interest	Phosphorus intake (1277 (mg))	Daily protein intake per day, g/kg				P for trend	P for interaction
		<0.8	0.8-1.0	1.0-1.2	>1.2		
L1	<median	1.058(0.876-1.278)	REF	0.887(0.675-1.166)	1.042(0.768-1.412)	0.4892	0.4764
	≥median	0.974(0.699-1.358)	REF	0.89(0.693-1.144)	0.802(0.623-1.032)	0.1217	
L2	<median	1.424(1.102-1.84)	REF	1.15(0.839-1.576)	1.836(1.336-2.525)	0.5251	0.8850
	≥median	1.315(0.904-1.913)	REF	1.061(0.726-1.55)	1.005(0.677-1.49)	0.5458	
L3	<median	1.324(0.967-1.812)	REF	0.945(0.658-1.356)	1.56(1.108-2.195)	0.9706	0.6015
	≥median	1.033(0.576-1.855)	REF	1.137(0.813-1.589)	1.143(0.812-1.608)	0.4791	
L4	<median	1.337(1.004-1.781)	REF	0.993(0.659-1.495)	1.617(1.124-2.325)	0.8637	0.5447
	≥median	1.474(0.887-2.449)	REF	1.175(0.815-1.695)	1.028(0.671-1.575)	0.4916	
All	<median	1.304(1.01-1.683)	REF	1.061(0.742-1.517)	1.749(1.284-2.384)	0.4267	0.5506
	≥median	1.545(1.098-2.176)	REF	1.165(0.845-1.606)	1.055(0.757-1.47)	0.3975	

Adjustment for age, gender, daily energy intake and body weight

Information Classification: General

Table 8 Relative risk of osteoporosis in lumbar spines in different level of protein diet (compared to 0.8-1.0 g per day/kg of daily protein intake) subgrouped by the **blood phosphorus concentration**

Region of interest	Blood phosphorus, (3.7(mg/dL))	Daily protein intake per day, g/kg				P for trend	P for interaction
		<0.8	0.8-1.0	1.0-1.2	>1.2		
L1	<median	0.987(0.801-1.216)	REF	1.07(0.81-1.412)	1.115(0.875-1.419)	0.3701	0.0463
	≥median	0.969(0.766-1.226)	REF	0.812(0.615-1.072)	0.813(0.621-1.064)	0.1062	
L2	<median	1.504(1.158-1.954)	REF	1.294(0.894-1.873)	1.554(1.108-2.178)	0.6269	0.3487
	≥median	1.218(0.94-1.578)	REF	1.036(0.729-1.472)	1.14(0.836-1.555)	0.7580	
L3	<median	1.336(1.018-1.754)	REF	1.332(0.987-1.798)	1.526(1.1-2.117)	0.2260	0.4751
	≥median	1.098(0.775-1.555)	REF	0.893(0.63-1.266)	1.122(0.835-1.509)	0.9784	
L4	<median	1.452(1.03-2.046)	REF	1.554(1.14-2.119)	1.508(1.054-2.157)	0.5140	0.1399
	≥median	1.13(0.816-1.565)	REF	0.83(0.55-1.252)	1.003(0.695-1.449)	0.4068	
All	<median	1.327(1.004-1.754)	REF	1.414(1.035-1.93)	1.479(1.085-2.015)	0.2550	0.4535
	≥median	1.211(0.916-1.602)	REF	0.965(0.666-1.399)	1.2(0.891-1.616)	0.9617	

Adjustment for age, gender, daily energy intake and body weight

Information Classification: General

Table 9. Relative risk of osteoporosis in lumbar spines in different level of protein diet (compared to 0.8-1.0 g per day/kg of daily protein intake) subgrouped by the **daily calcium and phosphorus intake level**

Region of interest	Daily calcium and phosphorus intake	Daily protein intake per day, g/kg				P for trend	P for interaction
		<0.8	0.8-1.0	1.0-1.2	>1.2		
L1	Ca<median; Ph<median	1.002(0.804-1.249)	REF	0.902(0.669-1.217)	1.034(0.725-1.474)	0.8737	0.9069
	Ca<median; Ph≥median	0.894(0.47-1.701)	REF	0.677(0.372-1.232)	0.668(0.409-1.09)	0.2214	
	Ca≥median; Ph<median	1.231(0.791-1.918)	REF	0.795(0.499-1.266)	0.991(0.509-1.93)	0.3024	
	Ca≥median; Ph≥median	1.013(0.713-1.441)	REF	0.975(0.738-1.288)	0.857(0.632-1.162)	0.2450	
L2	Ca<median; Ph<median	1.367(1.068-1.749)	REF	1.147(0.825-1.594)	1.971(1.378-2.818)	0.2385	0.5325
	Ca<median; Ph≥median	0.758(0.267-2.157)	REF	0.604(0.309-1.181)	0.847(0.47-1.525)	0.9582	
	Ca≥median; Ph<median	1.596(0.82-3.107)	REF	1.034(0.492-2.171)	1.219(0.582-2.551)	0.3736	
	Ca≥median; Ph≥median	1.619(1.055-2.485)	REF	1.271(0.823-1.963)	1.093(0.68-1.757)	0.5358	
L3	Ca<median; Ph<median	1.241(0.919-1.674)	REF	0.904(0.605-1.349)	1.641(1.099-2.451)	0.6756	0.3103
	Ca<median; Ph≥median	1.568(0.615-3.998)	REF	0.798(0.366-1.737)	1.106(0.586-2.085)	0.7685	
	Ca≥median; Ph<median	1.727(0.841-3.546)	REF	0.976(0.432-2.205)	1.091(0.495-2.401)	0.1786	
	Ca≥median; Ph≥median	0.8(0.346-1.849)	REF	1.274(0.842-1.928)	1.183(0.746-1.875)	0.2153	
L4	Ca<median; Ph<median	1.291(0.95-1.754)	REF	0.942(0.611-1.451)	1.645(1.111-2.436)	0.7467	0.6771
	Ca<median; Ph≥median	1.153(0.409-3.25)	REF	0.877(0.403-1.907)	1.028(0.534-1.982)	0.9473	
	Ca≥median; Ph<median	1.473(0.728-2.978)	REF	1.086(0.464-2.542)	1.386(0.614-3.129)	0.7760	
	Ca≥median; Ph≥median	1.616(0.831-3.143)	REF	1.329(0.89-1.985)	1.048(0.626-1.754)	0.4395	
All	Ca<median; Ph<median	1.249(0.951-1.639)	REF	1.053(0.725-1.53)	1.883(1.324-2.677)	0.1884	0.8853
	Ca<median; Ph≥median	1.153(0.499-2.663)	REF	0.813(0.438-1.509)	0.817(0.463-1.443)	0.4084	
	Ca≥median; Ph<median	1.518(0.807-2.857)	REF	0.989(0.503-1.945)	1.227(0.575-2.621)	0.4555	
	Ca≥median; Ph≥median	1.725(1.09-2.729)	REF	1.322(0.87-2.01)	1.171(0.744-1.843)	0.6614	

Adjustment for age, gender, daily energy intake and body weight

Information Classification: General

Table 10. Relative risk of osteoporosis in lumbar spines in different level of protein diet (compared to 0.8-1.0 g per day/kg of daily protein intake) subgrouped by the **blood phosphorus and phosphorus concentration level**

Region of interest	Blood phosphorus and phosphorus concentration level	Daily protein intake per day, g/kg				P for trend	P for interaction
		<0.8	0.8-1.0	1.0-1.2	>1.2		
L1	Ca<median; Ph<median	1.024(0.775-1.353)	REF	1.012(0.681-1.504)	1.021(0.754-1.382)	0.9973	0.2894
	Ca<median; Ph>=median	0.827(0.576-1.188)	REF	0.826(0.539-1.264)	0.777(0.543-1.111)	0.5851	
	Ca≥median; Ph<median	0.962(0.709-1.304)	REF	1.204(0.812-1.786)	1.283(0.882-1.864)	0.1798	
	Ca≥median; Ph≥median	1.07(0.831-1.379)	REF	0.822(0.565-1.194)	0.87(0.645-1.172)	0.0901	
L2	Ca<median; Ph<median	1.8(1.234-2.627)	REF	1.678(0.974-2.893)	1.726(1.102-2.703)	0.7155	0.7184
	Ca<median; Ph≥median	1.009(0.677-1.503)	REF	1.107(0.726-1.689)	1.065(0.686-1.654)	0.7421	
	Ca≥median; Ph<median	1.253(0.824-1.907)	REF	0.963(0.578-1.604)	1.394(0.797-2.438)	0.7161	
	Ca≥median; Ph≥median	1.399(1.028-1.905)	REF	0.992(0.603-1.632)	1.232(0.87-1.744)	0.5658	
L3	Ca<median; Ph<median	1.724(1.094-2.716)	REF	1.72(0.936-3.162)	1.564(0.912-2.684)	0.9276	0.8143
	Ca<median; Ph≥median	1.019(0.599-1.733)	REF	1.123(0.637-1.981)	1.191(0.707-2.006)	0.5198	
	Ca≥median; Ph<median	1.025(0.614-1.71)	REF	1.012(0.568-1.804)	1.537(0.807-2.927)	0.1809	
	Ca≥median; Ph≥median	1.139(0.729-1.781)	REF	0.765(0.465-1.258)	1.118(0.766-1.631)	0.7644	
L4	Ca<median; Ph<median	2.247(1.412-3.574)	REF	2.5(1.39-4.497)	1.892(1.097-3.262)	0.9384	0.7894
	Ca<median; Ph≥median	0.807(0.472-1.381)	REF	0.851(0.494-1.464)	0.905(0.501-1.635)	0.8094	
	Ca≥median; Ph<median	0.959(0.619-1.485)	REF	0.96(0.544-1.695)	1.288(0.73-2.275)	0.3413	
	Ca≥median; Ph≥median	1.408(0.972-2.04)	REF	0.837(0.526-1.332)	1.151(0.76-1.744)	0.2826	
All	Ca<median; Ph<median	1.853(1.212-2.834)	REF	2.074(1.195-3.598)	1.777(1.104-2.86)	0.5575	0.8531
	Ca<median; Ph≥median	0.88(0.564-1.372)	REF	1.048(0.681-1.613)	0.944(0.602-1.479)	0.7490	
	Ca≥median; Ph<median	0.951(0.627-1.443)	REF	0.926(0.559-1.534)	1.257(0.688-2.298)	0.3661	
	Ca≥median; Ph≥median	1.547(1.081-2.215)	REF	0.901(0.533-1.525)	1.485(1.066-2.069)	0.8634	

Adjustment for age, gender, daily energy intake and body weight

Information Classification: General

Table 11 Relative risk of osteoporosis in lumbar spines in different level of protein diet (compared to 0.8-1.0 g per day/kg of daily protein intake) subgrouped by the **daily vitamin D intake level**

Region of interest	Vitamin D Intake (2007-2010) (3.3 (mcg))	Daily protein intake per day, g/kg				P for trend	P for interaction
		<0.8	0.8-1.0	1.0-1.2	>1.2		
L1	<median	0.922(0.729-1.166)	REF	0.958(0.705-1.304)	1.031(0.723-1.469)	0.5677	0.3180
	≥median	1.034(0.762-1.404)	REF	1.059(0.703-1.596)	1.148(0.851-1.55)	0.4613	
L2	<median	1.014(0.692-1.485)	REF	1.111(0.739-1.67)	1.489(0.961-2.308)	0.0302	0.1890
	≥median	1.785(1.289-2.473)	REF	1.209(0.779-1.876)	1.375(0.939-2.014)	0.5279	
L3	<median	0.906(0.619-1.328)	REF	0.799(0.491-1.302)	1.344(0.893-2.022)	0.1372	0.4835
	≥median	1.286(0.859-1.926)	REF	1.335(0.839-2.123)	1.431(0.847-2.419)	0.3636	
L4	<median	0.949(0.647-1.393)	REF	0.706(0.372-1.338)	1.279(0.789-2.073)	0.4044	0.8782
	≥median	1.42(1.018-1.979)	REF	1.615(1.013-2.577)	1.258(0.802-1.974)	0.9631	
All	<median	0.95(0.663-1.36)	REF	0.84(0.543-1.3)	1.262(0.854-1.866)	0.2394	0.8531
	≥median	1.394(1.026-1.894)	REF	1.417(0.922-2.179)	1.375(0.976-1.939)	0.6193	

Adjustment for age, gender, daily energy intake and body weight

Information Classification: General

Table 12. Relative risk of osteoporosis in lumbar spines in different level of protein diet (compared to 0.8-1.0 g per day/kg of daily protein intake) subgrouped by the **blood vitamin D concentration**

Region of interest	Blood Vitamin D Level (62 (nmol/L))	Daily protein intake per day, g/kg				P for trend	P for interaction
		<0.8	0.8-1.0	1.0-1.2	>1.2		
L1	<median	0.947(0.768-1.168)	REF	1.094(0.845-1.415)	1.139(0.92-1.409)	0.0815	0.2627
	≥median	0.981(0.785-1.225)	REF	0.823(0.594-1.141)	0.744(0.553-1.001)	0.0487	
L2	<median	1.067(0.837-1.36)	REF	1.021(0.739-1.41)	1.253(0.903-1.737)	0.3586	0.0220
	≥median	1.729(1.312-2.278)	REF	1.372(0.925-2.035)	1.32(0.912-1.91)	0.3273	
L3	<median	1.119(0.857-1.463)	REF	1.056(0.793-1.406)	1.231(0.891-1.699)	0.5268	0.6290
	≥median	1.439(0.994-2.085)	REF	1.215(0.802-1.842)	1.338(0.944-1.895)	0.9155	
L4	<median	1.278(0.964-1.694)	REF	1.181(0.861-1.621)	1.262(0.872-1.827)	0.9335	0.8756
	≥median	1.337(0.908-1.969)	REF	1.11(0.698-1.766)	1.104(0.764-1.594)	0.4369	
All	<median	1.1(0.854-1.417)	REF	1.116(0.801-1.555)	1.276(0.94-1.731)	0.3297	0.3958
	≥median	1.558(1.164-2.086)	REF	1.317(0.849-2.043)	1.357(1.006-1.831)	0.6812	

Adjustment for age, gender, daily energy intake and body weight

Information Classification: General

Table 13. Femoral BMD based on CKD and non-CKD controls

Region of interest		Protein intake per day/kg				P for trend	P for interaction
		<0.8	0.8-1.0	1.0-1.2	>1.2		
Femur neck	Non-CKD[#]	0.834±0.003	0.837±0.003	0.836±0.004	0.843±0.003	0.0322	0.8552
	CKD	0.759±0.014	0.728±0.014	0.754±0.022	0.757±0.024	0.9222	
Trochanter	Non-CKD	0.731±0.003	0.738±0.003	0.738±0.004	0.741±0.003	0.0036	0.2874
	CKD	0.693±0.012	0.68±0.022	0.71±0.025	0.73±0.025	0.2750	
Intertrochanter	Non-CKD	1.143±0.004	1.153±0.004	1.155±0.005	1.157±0.004	0.0080	0.0317
	CKD	1.065±0.015	1.052±0.02	1.119±0.037	1.116±0.034	0.1372	
Total femur	Non-CKD	0.969±0.003	0.977±0.003	0.978±0.005	0.981±0.003	0.0039	0.1103
	CKD	0.907±0.013	0.892±0.018	0.939±0.03	0.941±0.028	0.2553	

Adjust for age, gender, energy, weight

[#]CKD: chronic kidney disease

Data presented as mean±SD

Published data by Chia-Lin Lee and Shang-Feng Tsai. Clin Nutr. 2020 Nov;39(11):3497-3503.

Information Classification: General

Table 14. Femoral T scores compared between CKD patients and controls

Region of interest		Protein intake per day/kg				P for trend	P for interaction
		<0.8	0.8-1.0	1.0-1.2	>1.2		
Femur neck	Non-CKD#	-0.48±0.024	-0.456±0.022	-0.468±0.032	-0.414±0.023	0.0461	0.7108
	CKD	-1.154±0.107	-1.412±0.109	-1.179±0.173	-1.17±0.179	0.9457	
Trochanter	Non-CKD	-0.153±0.024	-0.084±0.03	-0.09±0.035	-0.061±0.024	0.0063	0.2226
	CKD	-0.624±0.11	-0.743±0.196	-0.463±0.234	-0.301±0.23	0.2843	
Intertrochanter	Non-CKD	-0.06±0.026	0.002±0.024	0.013±0.035	0.025±0.025	0.0129	0.0208
	CKD	-0.702±0.091	-0.804±0.122	-0.355±0.241	-0.4±0.214	0.1517	
Total femur	Non-CKD	-0.174±0.025	-0.113±0.025	-0.113±0.034	-0.085±0.024	0.0066	0.0766
	CKD	-0.784±0.096	-0.911±0.128	-0.538±0.23	-0.541±0.208	0.2675	

Adjust for age, gender, energy, and body weight

#CKD: chronic kidney disease

Data presented as mean±SD

Published data by Chia-Lin Lee and Shang-Feng Tsai. Clin Nutr. 2020 Nov;39(11):3497-3503.

Information Classification: General

Table 15. Risk of osteoporosis by daily protein intake

Region of interest		Protein intake per day/kg				P for trend
		<0.8	0.8-1.0	1.0-1.2	>1.2	
Femur neck	Non-CKD#	REF	0.675(0.416-1.095)	0.736(0.44-1.232)	0.673(0.361-1.256)	0.2397
	CKD	REF	1.4(0.255-7.701)	0.459(0.091-2.314)	1.508(0.208-10.937)	0.8607
Trochanter	Non-CKD	REF	1.152(0.632-2.1)	0.837(0.443-1.581)	0.782(0.39-1.569)	0.3649
	CKD	REF	0.18(0.002-15.675)	0.186(0.037-0.942)	0.747(0.081-6.843)	0.6665
Intertrochanter	Non-CKD	REF	0.476(0.296-0.765)	0.546(0.258-1.154)	0.481(0.276-0.837)	0.0227
	CKD	REF	1.455(0.19-11.13)	0.359(0.032-4.054)	0.451(0.021-9.716)	0.5693
Total femur	Non-CKD	REF	0.567(0.354-0.908)	0.413(0.221-0.772)	0.49(0.28-0.858)	0.0063
	CKD	REF	0.32(0.015-6.897)	0.219(0.041-1.172)	0.43(0.038-4.884)	0.4016

Adjust for age, gender, energy, and body weight

#CKD: chronic kidney disease

Data presented as odds ratio (95% confidence intervals for odds ratios)

Published data by Chia-Lin Lee and Shang-Feng Tsai. Clin Nutr. 2020 Nov;39(11):3497-3503.

Information Classification: General

Table 16. Baseline characteristics of population with CKD divided by different protein intake.

**Variables**	**CKD(+)**
N	638
Age (years old)	69.24(67.82-70.67)
Male, n (%)	310(42.85)
Body weight (kg)	80.19(78.53-81.85)
Body mass index (kg/m^2)	28.94(28.47-29.41)
Systolic blood pressure (mm Hg)	136.32(134.1-138.54)
Diastolic blood pressure, (mm Hg)	67.55(66.32-68.78)
Smoking, n (%)	331(48.94)
Cardiovascular disease, n (%)	82(11.7)
Diabetes, n (%)	248(32.83)
Total cholesterol, mg/dl	196.3(192.14-200.47)
HDL cholesterol, mg/dl	53.44(52.06-54.81)
Triglycerides, mg/dl	171.45(158.94-183.95)
Fasting plasma glucose, mg/dl	112.69(107.36-118.03)
HbA1c[&], %	5.95(5.86-6.05)
Blood total clacium, mg/dl	9.45(9.39-9.5)
Blood phosphorus, mg/dl	3.89(3.82-3.95)
Vitamin D level, nmol/L	66.7(64.04-69.36)
eGFR[@] (MDRD), mL/min/1.73 m^2	48.76(47.81-49.71)
eGFR[@](CKD-EPI), mL/min/1.73 m^2	48.18(47.17-49.19)
Protein intake per day, g/kg	0.9(0.86-0.94)
Calcium intake, mg per day	804.84(743.34-866.33)
Phosphorus intake, mg per day	1156.39(1089.28-1223.5)
Vitamin D (D2+D3) intake, mg per day	4.08(3.7-4.47)
Calorie intake, kcal/day/kg	22.52(21.56-23.47)
Calorie intake, kcal/day	1745.95(1653.91-1838)
% from carbohydrate	0.49(0.48-0.5)
% from fat	0.34(0.34-0.35)
% from protein	0.16(0.16-0.17)
L1 BMD	0.91(0.9-0.93)
L1 T score	-2.35(-2.5--2.2)
L2 BMD	1.01(0.99-1.03)
L2 T score	-1.58(-1.73--1.43)
L3 BMD	1.05(1.03-1.07)
L3 T score	-1.25(-1.4--1.1)
L4 BMD	1.06(1.04-1.07)
L4 T score	-1.15(-1.28--1.03)
All BMD	1.01(1-1.03)
All T score	-1.54(-1.68--1.41)

*CKD, chronic kidney disease; [&] HbA1c, glycated hemoglobin; [@] eGFR, estimated glomerular filtration rate; [#] BMD, bone mineral density.

Information Classification: General

Figure 1A Lumbar BMD and T score according to different level of daily protein intake divided by CKD or not. BMD in non-CKD group according to different protein intake in lumbar spines

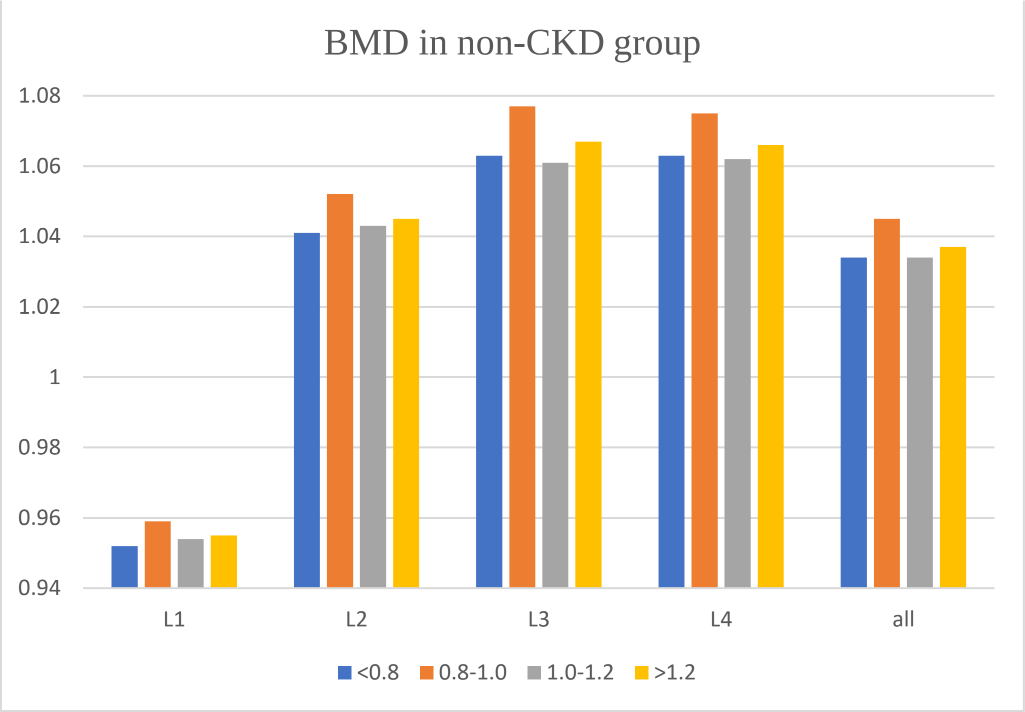

Figure 1B Lumbar BMD and T score according to different level of daily protein intake divided by CKD or not. BMD in CKD group according to different protein intake in lumbar spines.

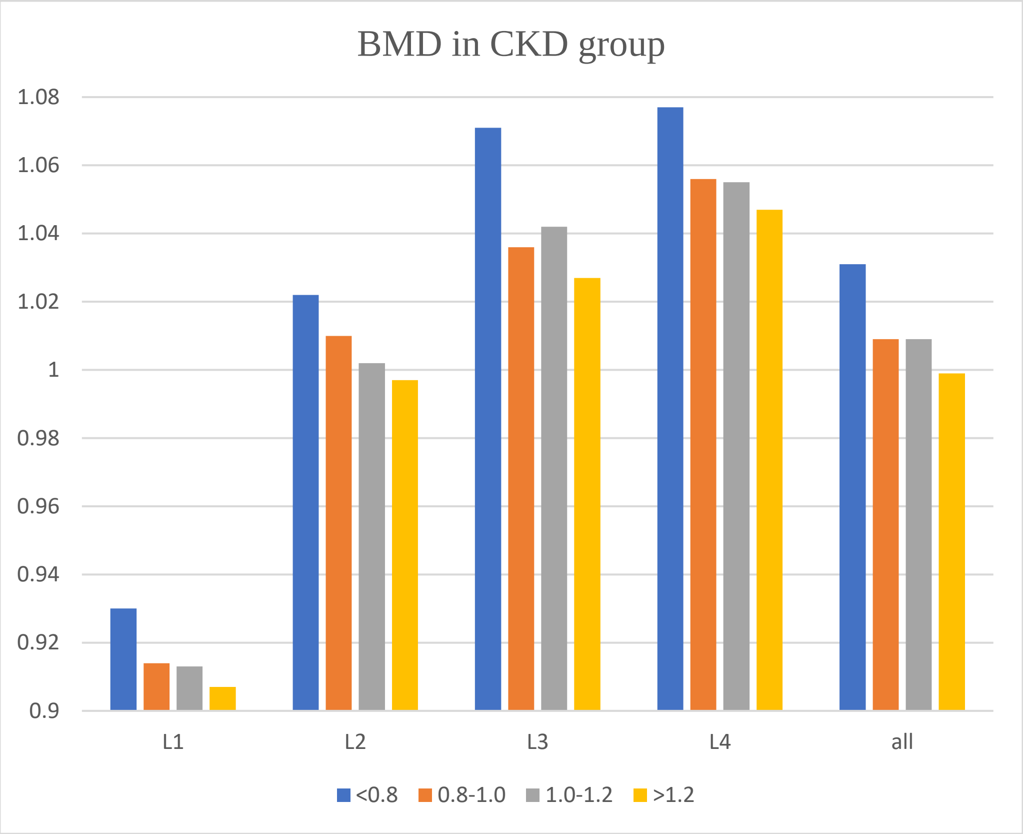

Information Classification: General

Figure 1C Lumbar BMD and T score according to different level of daily protein intake divided by CKD or not. T score in non-CKD group according to different protein intake in lumbar spines.

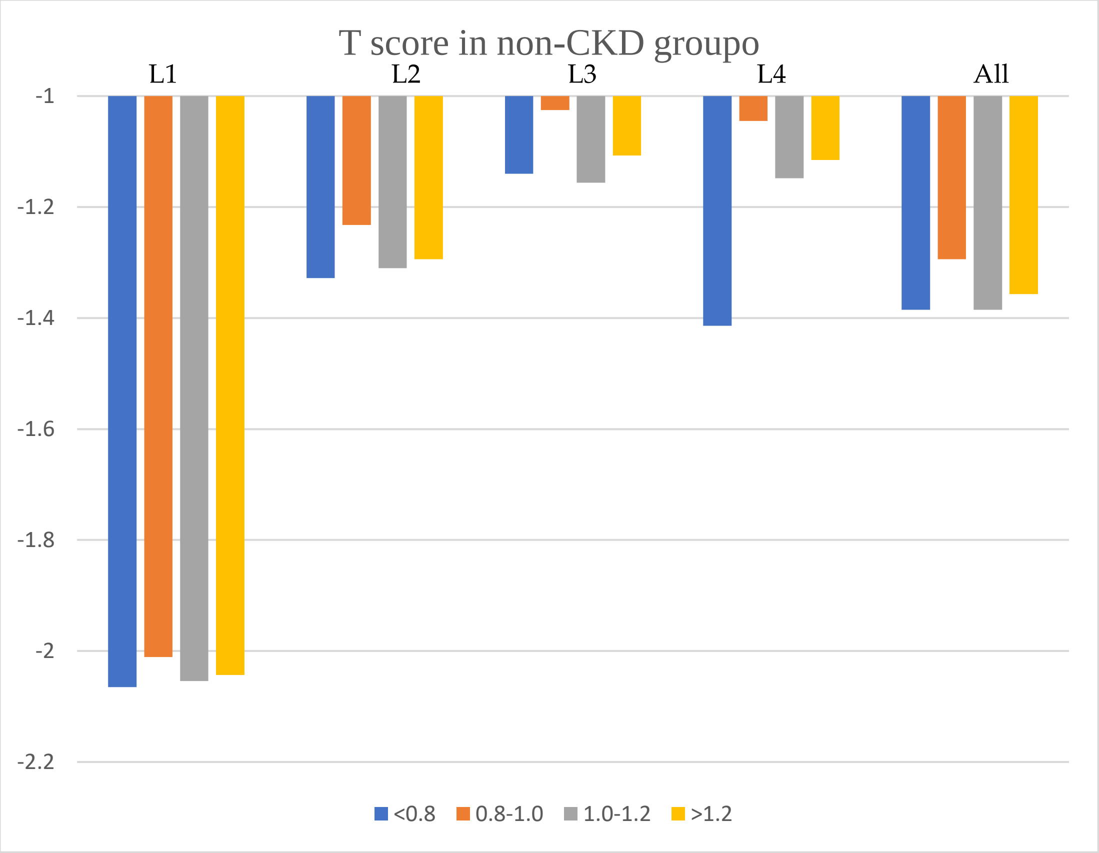

Figure 1D Lumbar BMD and T score according to different level of daily protein intake divided by CKD or not. T score in CKD group according to different protein intake in lumbar spines

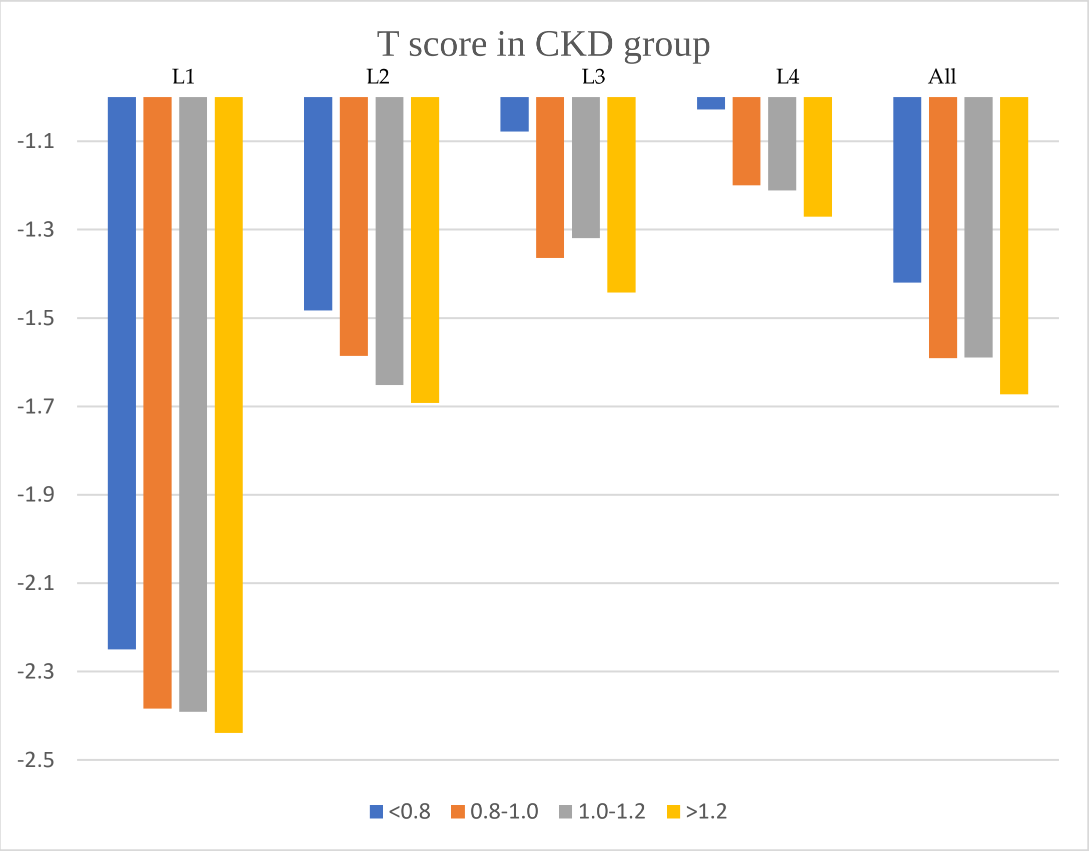

Figure 1E Lumbar BMD and T score according to different level of daily protein intake divided by CKD or not. Relative risk for osteoporosis in non-CKD group according to different protein intake in lumbar spines (reference: DPI: 0.8-1.0 g/day/kg).

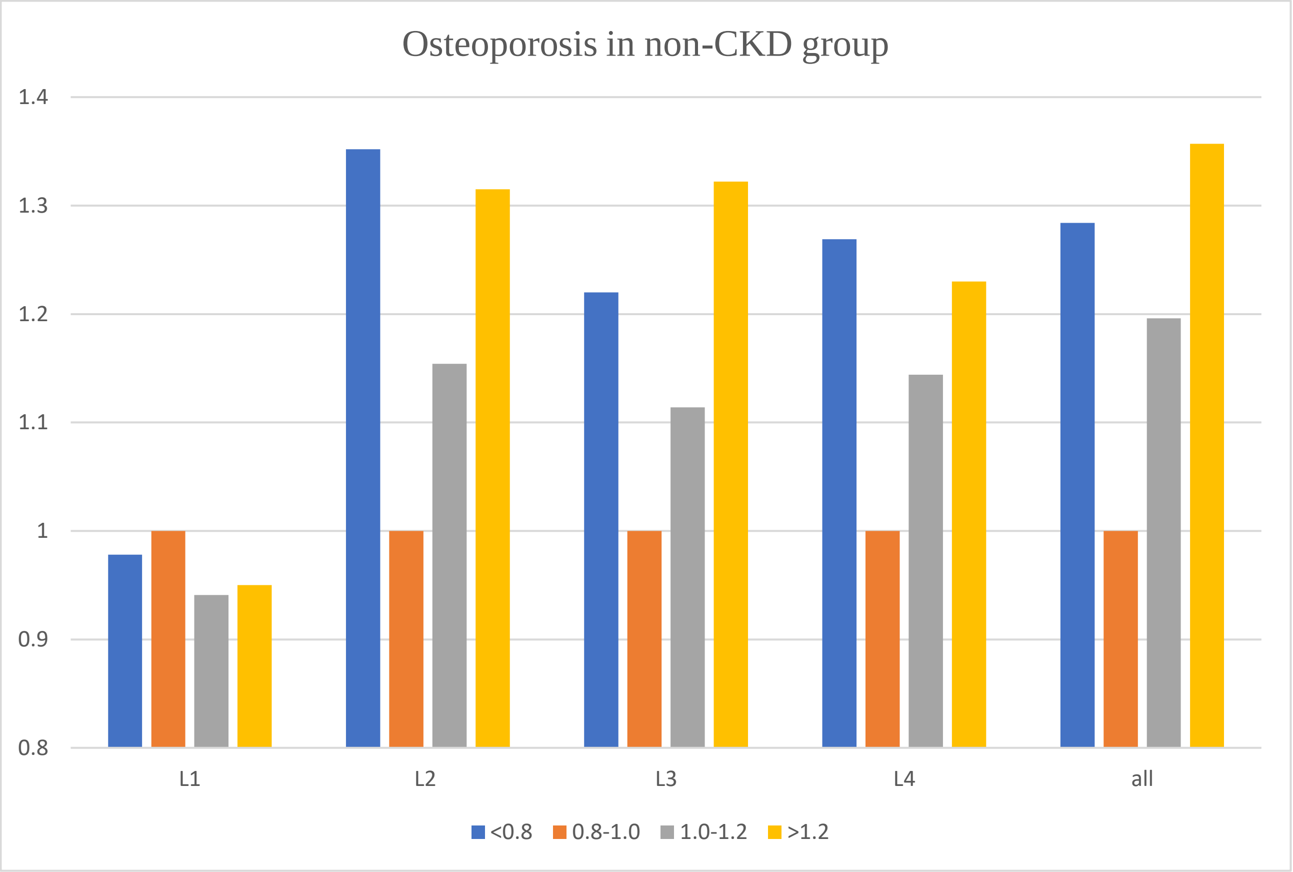

Figure 1F Lumbar BMD and T score according to different level of daily protein intake divided by CKD or not. Relative risk for osteoporosis in CKD group according to different protein intake in lumbar spines (reference: DPI: 0.8-1.0 g/day/kg).

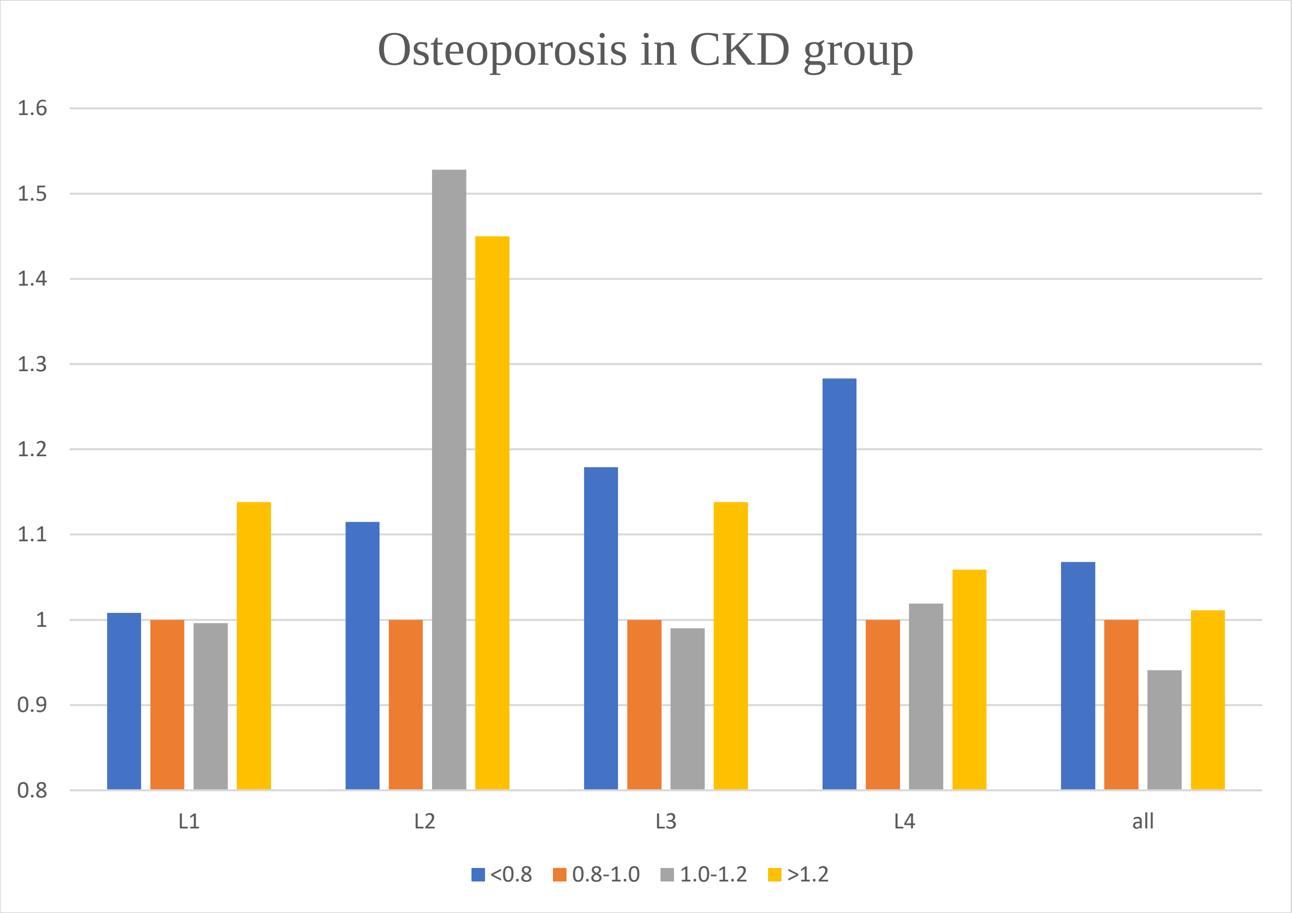

Information Classification: General

Information Classification: General
